# Effects of polarization mode dispersion on polarization-entangled photons generated via broadband pumped spontaneous parametric down-conversion

**DOI:** 10.1038/srep25846

**Published:** 2016-05-13

**Authors:** Hyang-Tag Lim, Kang-Hee Hong, Yoon-Ho Kim

**Affiliations:** 1 Department of Physics, Pohang University of Science and Technology (POSTECH), Pohang, 790-784, Korea

## Abstract

An inexpensive and compact frequency multi-mode diode laser enables a compact two-photon polarization entanglement source via the continuous wave broadband pumped spontaneous parametric down-conversion (SPDC) process. Entanglement degradation caused by polarization mode dispersion (PMD) is one of the critical issues in optical fiber-based polarization entanglement distribution. We theoretically and experimentally investigate how the initial entanglement is degraded when the two-photon polarization entangled state undergoes PMD. We report an effect of PMD unique to broadband pumped SPDC, equally applicable to pulsed pumping as well as cw broadband pumping, which is that the amount of the entanglement degradation is asymmetrical to the PMD introduced to each quantum channel. We believe that our results have important applications in long-distance distribution of polarization entanglement via optical fiber channels.

Photons are considered as one of the ideal candidates for practical quantum information processing^1^,^2^,^3^,^4^. This is mainly due to both of the facts that photon pairs generated via the spontaneous parametric down-conversion (SPDC) process are convenient and efficient resources for entanglement generation^5^,^6^,^7^,^8^, and that photons are robust against decoherence since they do not easily interact with the environment, which make it possible to distribute entanglement for long-distance communications.

There are various ways to pump the nonlinear crystal for generation of the two-photon states via the SPDC process. One way is using a narrowband continuous wave (CW) laser such as an ion laser for pumping the nonlinear crystal^9^,^10^,^11^. The two-photon state can also be prepared by using femto-second pulsed pump lasers^7^,^12^. In addition, the recent development of the multi-mode frequency diode laser enables two-photon generation via the CW broadband pumped SPDC process^13^,^14^,^15^. Due to the inexpensive price, compactness, and relative high-power of the laser (a few hundred milliwatt), the SPDC source using multi-mode diode lasers is considered as an ideal two-photon source for practical quantum information processing.

Among various degrees of freedom of photons, a photonic polarization qubit is widely used since polarization states can be easily manipulated with linear optical elements^2^,^16^. However, even though photonic polarization states are robust under most environmental conditions including free-space communication, they suffer from polarization mode dispersion (PMD) caused by the birefringence of media, when they propagate through optical fibers^17^. This effect results in decoherence, or degradation of entanglement^18^ during the distribution and storage of photons using optical fibers. Therefore, understanding the effect of PMD on polarization entanglement is necessary for finding ways to effectively distribute polarization entanglement for long-distance communication.

Previous researches have been devoted to understand the effect of the PMD on polarization entangled states generated via the SPDC process^19^,^20^,^21^,^22^. A theoretical study about PMD effect when the polarization entangled photons are distributed through optical fibers was reported in ref. 20. Experimental verification of the entanglement degradation when only one of the entangled photon pairs undergoes PMD has been reported^21^. Note that the polarization entangled states prepared via the pulsed SPDC are considered in refs 20 and 21. Furthermore, it has recently been experimentally demonstrated that narrowing frequency bandwidths of the photons is helpful for preserving more entanglement when each photon suffers from the same amounts of PMDs^22^. However, although ref. 22 provides insights about finding a way to suppress entanglement degradation due to PMD, the detailed analysis on how the photon’s frequency bandwidth affects the behavior of the entanglement degradation caused by PMD has not been studied. Moreover, there still has been no experimental demonstration that reveals the overall features of entanglement degradation when each photon undergoes different amount of PMD.

In this paper, we consider broadband pumped SPDC for generating polarization entangled photons and a realistic quantum communication scenario in which the initially prepared polarization entangled photons are distributed through optical fiber channels. We theoretically calculate the concurrence of the polarization entanglement after the initial entangled photon pairs undergo independent PMDs. Then, we experimentally demonstrate that the polarization entanglement from the broadband CW pumped SPDC experiences unreported PMD effects in that the amount of the entanglement degradation is asymmetrical to the PMD introduced to each quantum channel. Here, we emphasize that this result is equally applicable to pulsed pumping as well as CW broadband pumping. We believe that our results have important applications in long-distance distribution of polarization entanglement via optical fiber channels.

## Results

**Figure 1:**
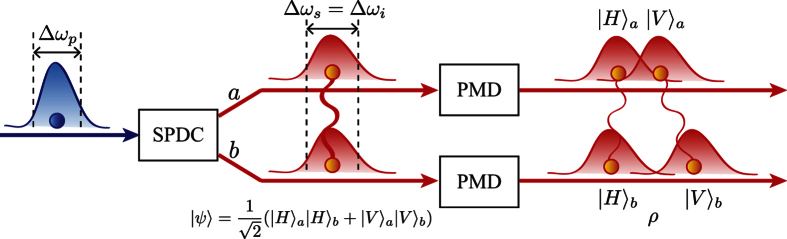
Schematic of the scenario. The pump photon is split into two down-converted photons (one is called the signal and the other is called the idler) via the broadband pumped SPDC process. Then, the down-converted photons are prepared as a two-photon polarization entangled state, and each photon undergoes independent PMD. Since the group velocities of the horizontal and vertical photons become different in birefringent medium, the distinguishabilities between two polarization components in each photon result in degradation of the polarization entanglement. Here the pump and the down-converted photons are correlated in a frequency mode due to the phase matching condition of the SPDC process. Since PMD is closely related to the frequency of photons, the polarization entanglement degradation due to PMD is influenced by frequency bandwidths of the pump (Δ*ω*_*p*_), the signal (Δ*ω*_*s*_), and the idler (Δ*ω*_*i*_) photons.

Here we consider the scenario that the two-photon polarization entangled state generated via the broadband pumped SPDC process undergoes independent PMD as shown in Fig. 1. The broadband pumped SPDC which we consider in this manuscript deals with a cw pump laser having broadband but all modes are incoherent. Note that, in general, the longitudinal (frequency) modes of a free-running cw lasers are incoherent. In order to prepare the polarization entangled state, a pair of two photons is generated from type-I non-collinear parametric down conversion process in *β*-BaB_2_O_4_ (BBO) crystal. When the multi-mode diode laser is used for pumping the nonlinear crystal, the two-photon state after transmitting through the interference filters (IFs) can be expressed in the following form^13^,^14^,


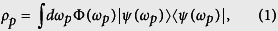


where *ω*_*p*_ is the frequency of the pump photon and the spectral power density function Φ (*ω*_*p*_) is given by


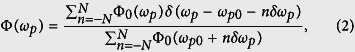


where *ω*_*p*0_ is the center frequency of the pump photon, *δω*_*p*_ is the mode spacing, *n* is the mode number, and the spectral profile of the pump Φ_0_ (*ω*_*p*_) is Gaussian,


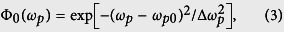


where Δ*ω*_*p*_ is the bandwidth of the pump photon. The monochromatic laser pumped SPDC two-photon state |*ψ*(*ω*_*p*_)〉 is written as^13^,^14^


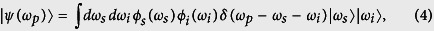


where 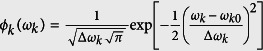 with *k* = *s* and *i* correspond to the filter transmission function of the signal and the idler photons, respectively, satisfying the normalization condition 
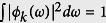
, and *ω*_*k*0_ and Δ*ω*_*k*_ are the center wavelength and the bandwidth of the IF, respectively. Since the IFs in the two optical paths are identical to each other, the frequency centers and bandwidths of the signal and the idler photons are the same; that is, *ω*_*s*0_ = *ω*_*i*0_ and Δ*ω*_*s*_ = Δ*ω*_*i*_. The subscript *s* and *i* refer to the states of the signal and the idler photons, respectively.

The polarization entangled photon pair can be prepared using the Hong-Ou-Mandel (HOM)^9^,^23^ interferometer and post-selection. We send the two photons into the HOM interferometer and post-select the case that each photon comes out from different output ports *a* and *b* of the HOM interferometer as depicted in Fig. 1 9,15. The post-selected state is the entangled state having the following form,


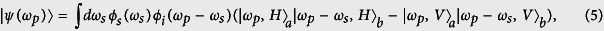


where *H* and *V* refer to the horizontal and vertical polarizations, respectively. The subscripts indicate the output modes of the interferometer.

In each optical path, PMD may come from birefringent optical elements such as PM fibers and quartz crystals. The origin of PMD is the difference of the refractive indices between two orthogonal polarization modes, which causes the difference in the amounts of phase shift between the two polarization modes. After each photon undergoes PMD in the mode *a* and *b*, the state evolves to


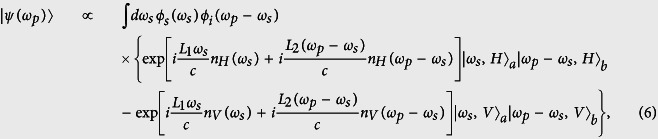


where *L*_1_ and *L*_2_ are the lengths of the birefringent material in the optical path *a* and *b*, and *n*_*H*_(*ω*) and *n*_*V*_(*ω*) refer to the refractive indices for horizontally and vertically polarized photons at frequency *ω*, respectively. The refractive indices of materials are dependent on the frequency of photons. Here we take an approximation on the refractive indices up to the first order in a frequency, so that 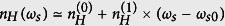, and 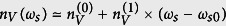, where 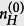 and 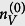 are the refractive index values at the frequency *ω*_*s*0_ and 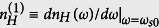 and 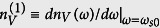 are the first order derivatives of the refractive indices at *ω*_*s*0_. *c* is the speed of light.

Since single photon detector cannot distinguish photon’s frequency, the final state is obtained by tracing out the frequency degree of freedom in Eq. (6). The detailed information on how to evaluate concurrence^24^ of the final state is provided in the Methods section.

**Figure 2:**
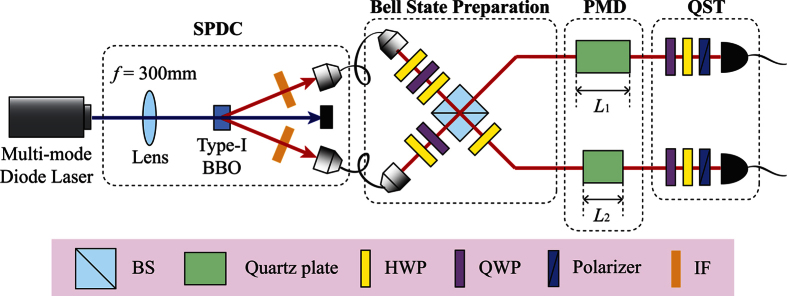
Experimental setup. The experimental setup for verifying the PMD effect on the polarization entangled state generated via the SPDC process. Two-photon state is prepared from type-I non-collinear SPDC process with a multi-mode diode laser. The HOM interferometer with additional wave plates is used to prepare the two-photon polarization entangled state. Various amounts of PMD are introduced by different lengths of birefringent quartz plate in each optical path. The concurrence of the final two-photon polarization state is analyzed by using quantum state tomography (QST). BS: beam splitter, HWP: half-wave plate, QWP: quarter-wave plate, IF: interference filter.

The experimental setup is schematically shown in Fig. 2 and is composed of four parts: SPDC, Bell state preparation, PMD, and quantum state tomography (QST). Detailed information about the experimental setup is described in the Methods section.

**Figure 3:**
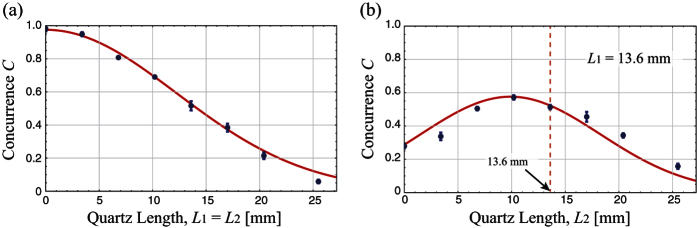
Experimental results: concurrence as the function of the lengths of the quartz plates. The horizontal axes represent the length of quartz plate, which corresponds to the amount of PMD. The vertical axes represent the concurrence of the final state. (**a**) The concurrence values when the same lengths of quartzes are introduced on both optical paths. (**b**) The concurrence values when the quartz length of one optical path is fixed as *L*_1_ = 13.6 mm. The Black dots are the experimental data and the vertical error bars represent one standard deviation. The red lines are the theoretical curves with fitting parameters Δ*ω*_*p*_ and Δ*ω*_*s*_.

Figure 3(a) shows the concurrence of the situation in which the same amounts of PMD are introduced on each optical path, and Fig. 3(b) shows the concurrence of the case that the amount of PMD on one path is fixed (with *L*_1_ = 13.6 mm), while the PMD on the other optical path is varying. In the former case the concurrence decreases gradually as the length of the quartz plates increases. In the latter case, however, the concurrence does not monotonically decreases as the amount of PMD on one photon increases. Moreover, the concurrence maximum value is obtained when *L*_2_ is around 10 mm, which is clearly smaller than *L*_1_ = 13.6 mm, i.e., *L*_2_ < *L*_1_. We conclude from this result that when only one of the polarization entangled photon undergoes PMD, making the other photon undergo the smaller amount of the PMD can effectively preserve more entanglement. For instance, when only one photon undergoes PMD (*L*_1_ = 13.6 mm), the concurrence *C*(0*L*) is more than two times smaller than its maximum value in Fig. 3(b). In addition, the maximum concurrence value is about 10% larger than the case that each photon undergoes the same amount of the PMD effect.

To confirm the validity of our theoretical calculation, we estimate the frequency bandwidths of the pump and the down-convert photons from the experimental data. (See the Method section for the concurrence of the final state.). We used the mode spacing of the pump photon as *δω*_*p*_ = 3.24 × 10^11^ Hz, which corresponds to the mode spacing of the wavelength Δ*λ* = 0.0282 nm^13^. The fitting values are evaluated to be Δ*ω*_*p*_ = (3.95 ± 0.10) × 10^12^ Hz and Δ*ω*_*s*_ = (7.37 ± 0.28) × 10^12^ Hz. The red lines in Fig. 3 are the fitting curves with these values. From the HOM interference signal, we obtained the bandwidth of the signal photons as 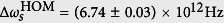, which is close to the estimated values from the result of Fig. 3.

**Figure 4:**
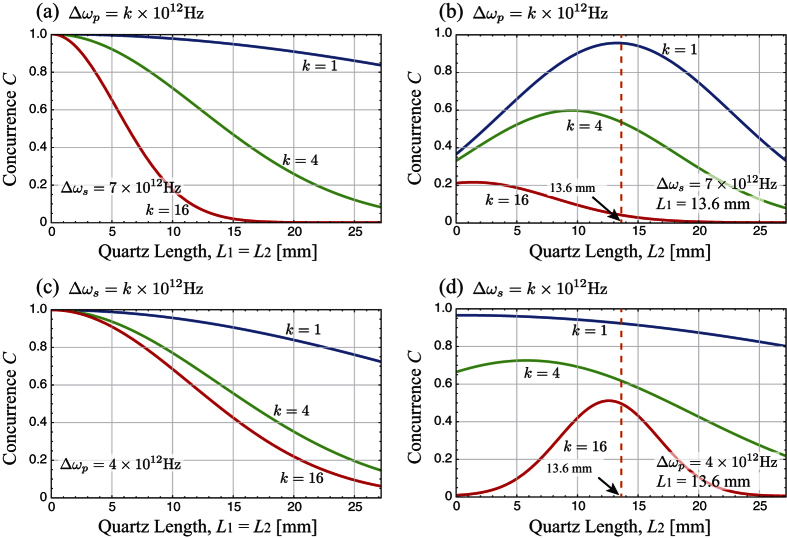
Theoretical simulation of the concurrence. Concurrence for various conditions on the frequency bandwidths of the pump and the down-converted photons. Here the signal and the idler photons have the same frequency bandwidth Δ*ω*_*s*_. (**a**,**b**) describe the effect of pump photon’s bandwidth Δ*ω*_*p*_ on the concurrence when Δ*ω*_*s*_ = 7 × 10^12^ Hz. On the other hand, (**c**,**d**) show the concurrence dependency on Δ*ω*_*s*_ with fixed Δ*ω*_*p*_ = 4 × 10^12^ Hz. (**a**,**c**) correspond the case that the identical amounts of PMD are introduced on both optical paths, while (**b**,**d**) refer to the case that different amounts of PMD are introduced. Note that the vertical dotted lines in (**b**,**d**) correspond to the length of the quartz crystal (*L*_1_ = 13.6 mm) which the photon in the optical path 1 passes through.

Theoretical simulation results of the pump and the down-converted photon’s frequency bandwidths effects on the concurrence are graphically shown in Fig. 4. The concurrence degradation behavior is depicted by changing either Δ*ω*_*p*_ or Δ*ω*_*s*_ values. Figure 4(a,b) show the effect of the pump photon’s frequency bandwidth, while Fig. 4(c,d) describe the down-converted photon’s frequency bandwidth effect. Δ*ω*_*p*_ = 4 × 10^12^ Hz and Δ*ω*_*s*_ = 7 × 10^12^ Hz are chosen based on the realistic values from our experiment.

Figure 4(a,c) represent the case that the amounts of PMD applied on both photons are the same, showing that concurrence degradation increases as the bandwidths of the pump, the signal, the idler photons increase. Interestingly, the concurrence appears to be more sensitive to the increments of Δ*ω*_*p*_ than the increments of Δ*ω*_*s*_.

Figure 4(b,d) show the concurrence tendency where the amount of the PMD on one optical path is varied while the amount of the PMD on the other optical path is fixed as *L*_1_ = 13.6 mm. For a fixed bandwidth Δ*ω*_*s*_, the concurrence has its maximum value near at *L*_2_ = 13.6 mm when Δ*ω*_*p*_ is small, while its maximum point asymptotically approaches *L*_2_ = 0 as Δ*ω*_*p*_ increases. In contrast, for a fixed Δ*ω*_*p*_ value, the quartz length giving the concurrence maximum approaches to *L*_2_ = *L*_1_ as Δ*ω*_*s*_ increases.

## Discussion

A broadband CW pumped SPDC using an inexpensive and compact frequency multi-mode diode laser is an attractive practical resource for entangled state generation. In this work, we have considered a realistic quantum communication scenario in which the initial two-photon polarization entangled states are distributed through optical fiber channels. Since PMD in the birefringent optical element leads to the degradation of the polarization entanglement, understanding PMD effects on polarization entangled state is one of the important issues in practical quantum information such as long distance communication. We have theoretically and experimentally investigated how the frequency bandwidths of the pump and the down-converted photons affect the two-photon polarization entanglement degradation caused by PMD. From the results, we find that the amount of the entanglement degradation is asymmetrical to the PMD introduced to each quantum channel, which is an unique effect of the broadband pumping, equally applicable to pulsed pumping as well as CW broadband puming. This is an intriguing result since the same amount of the PMD is introduced on each photon for preserving entanglement against PMD effects in ref. 22. Therefore, we believe that our results can give an insight on the degradation of entanglement caused by PMD in long-distance communications via optical fiber channels and are helpful for overcoming decoherence effect on the polarization entanglement.

## Methods

### Concurrence of the final state

In order to calculate the final two-photon polarization density matrix, we firstly trace out the frequency mode of the final state |*ψ*(*ω*_*p*_)〉 in Eq. (6). Then, the two-photon polarization density matrix *ρ*(*ω*_*p*_) = Tr_*ω*_[|*ψ*(*ω*_*p*_)〉〈*ψ*(*ω*_*p*_)|] is evaluated to be


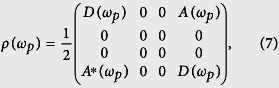


where A*(*ω*_*p*_) is the complex conjugate of *A*(*ω*_*p*_), and *D*(*ω*_*p*_) and *A*(*ω*_*p*_) have the following form,


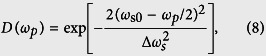


and


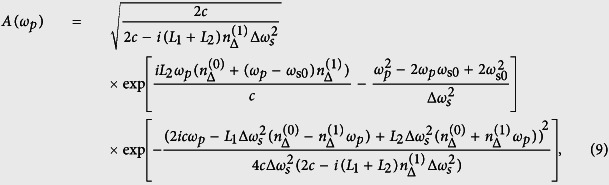


respectively. Here 
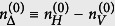
 and 
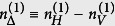
. After integrating over the pump photon spectrum, the concurrence of the total multi-mode final state can be calculated. The concurrence of the two-qubit density matrix is found to be *C*(*ρ*) = max[0, *λ*_1_ − *λ*_2_ − *λ*_3_ − *λ*_4_] where *λ*_*n*_’s are the eigenvalues of the matrix 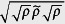 with 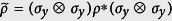 in decreasing order and ρ* is the complex conjugate of *ρ*^24^. In our case, the final density matrix has only a few non-vanishing terms, the concurrence of the final state can be calculated to be as follows:


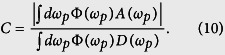


### State preparation

A 100 mW multi-mode laser with central wavelength of 405 nm is used to pump a non-linear crystal. Then, the pump photon is probabilistically split into the signal and the idler photons via type-I non-collinear SPDC process in 6-mm-thick BBO crystal. The down-converted photons are frequency filtered by the IFs with 810 nm central frequency and 5 nm full width half maximum (FWHM) bandwidth. By post-selecting the case that one photon is in each output modes of the HOM interferometer, the output state is prepared as 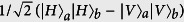 in a polarization mode^9^,^15^.

### Polarization mode dispersion

After the frequency anti-correlated polarization Bell state is prepared, the quartz plates are introduced to apply PMD effect on each down-converted photon^25^. With vertically aligned optic axis, the quartz plate has the refractive indices of 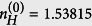 and 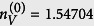 in the 0th order, and 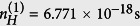 and 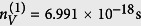 in the 1st order expansion in a frequency mode^26^. We perform the experiments for eight different values of PMD on each optical path. Various amounts of PMD are implemented by using a set of different quartz plates whose lengths are 2*L*, 4*L*, 6*L*, 8*L*, 10*L*, 12*L* and 15*L*, where *L* = 1.7 mm. Furthermore, we perform the experiments for the case that one photon undergoes a fixed amount of PMD (*L*_1_ = 8*L* = 13.6 mm), while changing the amount of PMD that the other photon experiences. Finally, the concurrence of the final state after undergoing PMD is analyzed by using two-qubit quantum state tomography (QST).

## Additional Information

**How to cite this article**: Lim, H.-T. *et al*. Effects of polarization mode dispersion on polarization-entangled photons generated via broadband pumped spontaneous parametric down-conversion. *Sci. Rep*. **6**, 25846; doi: 10.1038/srep25846 (2016).
